# Supplementary material for: Morphology of Composite Fe@Au Submicron Particles, Produced with Ultrasonic Spray Pyrolysis and Potential for Synthesis of Fe@Au Core–Shell Particles
Source: Materials (Basel). 2019 Oct 12;12(20):3326. doi: 10.3390/ma12203326 (PMC6829231; doi:10.3390/ma12203326)
Supplement: Supplementary file 1 [file materials-12-03326-s001.pdf]

materials

Supplementary materials of

Article

Morphology of Composite Fe@Au Submicron Particles, Produced with Ultrasonic Spray Pyrolysis and Potential for Synthesis of Fe@Au Core–Shell Particles

Peter Majerič [1,2,*], Darja Feizpour [3], Bernd Friedrich [4], Žiga Jelen [1,5], Ivan Anžel [1] and Rebeka Rudolf [1,2]

[1] Faculty of Mechanical Engineering, University of Maribor, Smetanova ulica 17, 2000 Maribor, Slovenia; ivan.anzel@um.si (I.A.); rebeka.rudolf@um.si (R.R.)
[2] Zlatarna Celje d.o.o., Kersnikova 19, 3000 Celje, Slovenia
[3] Institute of Metals and Technology, Lepi pot 11, 1000 Ljubljana, Slovenia; darja.feizpour@imt.si
[4] IME Process Metallurgy and Metal Recycling, RWTH Aachen University, Intzestrasse 3, 52065 Aachen, Germany; bfriedrich@ime-aachen.de
[5] Plastika Skaza d.o.o., Selo 22, 3320 Velenje, Slovenia; z.jelen@um.si
* Correspondence: peter.majeric@um.si or peter.majeric@zlatarnacelje.si

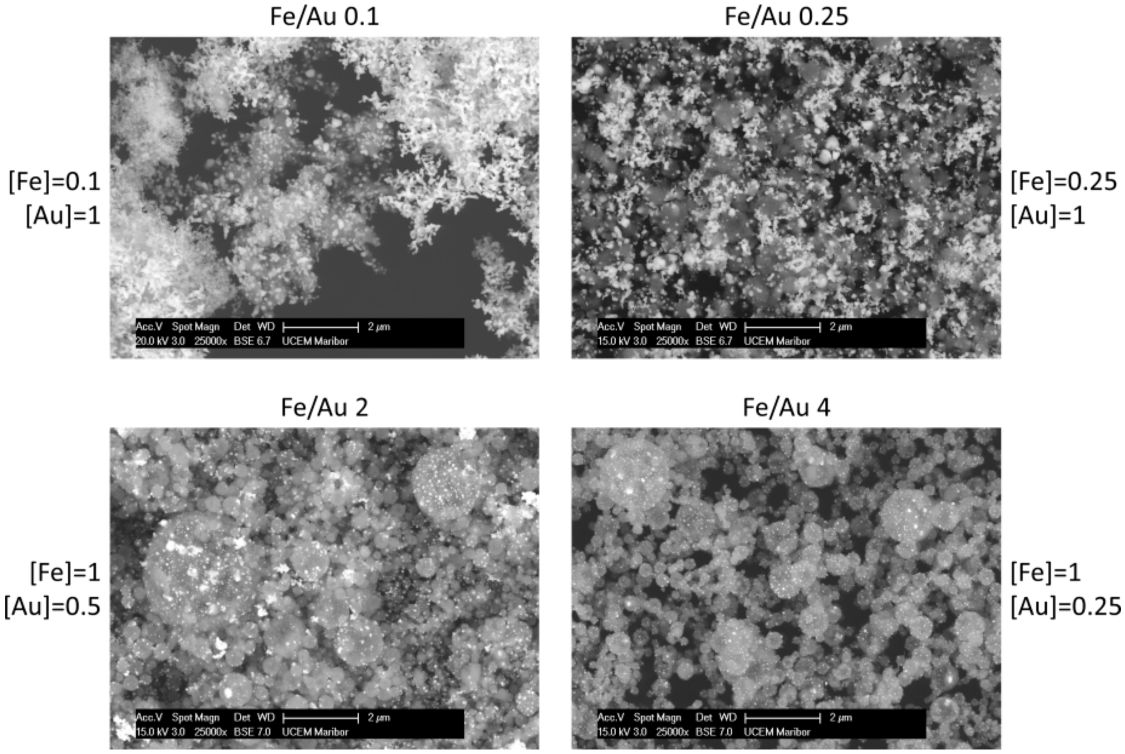

Figure S1. Low magnification SEM images of the Fe@Au particles produced in experiments with different Fe/Au concentration ratios. Concentrations are in g/L.

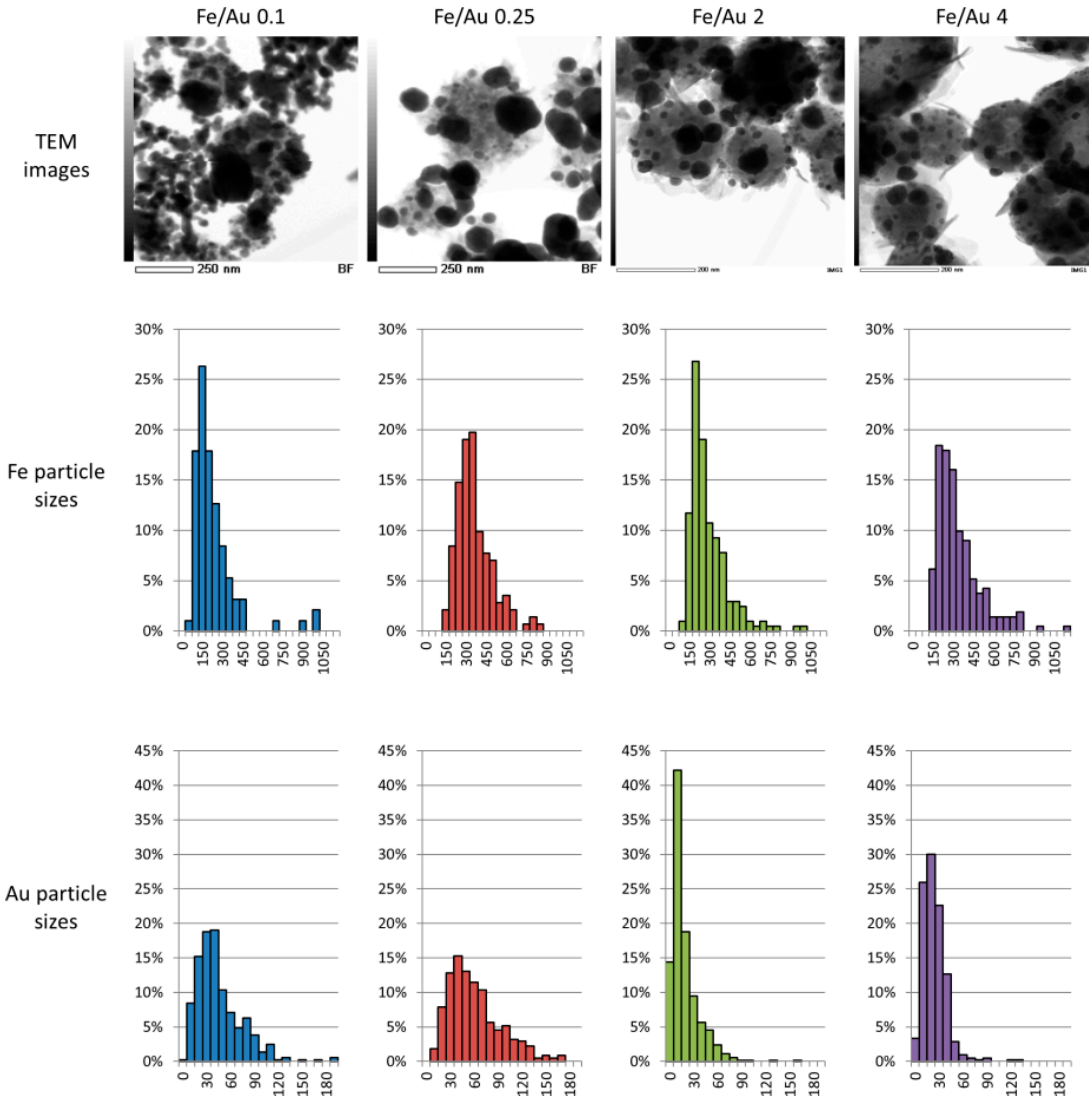

Figure S2. Bright Field TEM images corresponding to EDX images in Figure 4, with size distributions of Fe oxide particles and AuNPs, measured from TEM and SEM images. The vertical axis in the size distributions is the number frequency in %, the horizontal axis is size in nm.

Table S1. Fe oxide and Au nanoparticle size measurements: mean value (MEAN), Standard Deviation (SD), maximum particle size (MAX), minimum particle size (MIN) and number of particles measured (n). Values are in nm.

**Fe Oxide Particle Sizes**				
	**Fe/Au 0.1**	**Fe/Au 0.25**	**Fe/Au 2**	**Fe/Au 4**
MEAN	258.9	390.47	336.02	373.77
SD	170.48	132.18	229.94	186.35
MAX	1033.74	853.74	2473.44	1313.52
MIN	99.77	152.65	109.40	169.03
n	95	142	205	212
**Au Particle Sizes**				
	**Fe/Au 0.1**	**Fe/Au 0.25**	**Fe/Au 2**	**Fe/Au 4**
MEAN	45.47	67.47	24.01	26.49
SD	33.72	36.38	20.66	16.99
MAX	306.90	242.85	232.35	136.14
MIN	8.58	14.12	398	6.13
n	368	445	548	420
